# Vitamin D Status as an Important Predictor of Preterm Birth in a Cohort of Black Women

**DOI:** 10.3390/nu15214637

**Published:** 2023-11-01

**Authors:** Jennifer Woo, Thomas Guffey, Rhonda Dailey, Dawn Misra, Carmen Giurgescu

**Affiliations:** 1 College of Nursing, Texas Woman’s University, Dallas, TX 75235, USA; 2 Greene Center for Reproductive Biology, UT Southwestern, Dallas, TX 75390, USA; 3 Center for Research Design and Analysis, Texas Woman’s University, Denton, TX 76204, USA; tguffey@twu.edu; 4 Department of Family Medicine and Public Health Sciences, Wayne State University, Detroit, MI 48202, USA; rdailey@med.wayne.edu; 5 Department of Epidemiology and Biostatistics, Michigan State University, East Lansing, MI 48823, USA; misradaw@msu.edu; 6 College of Nursing, University of Central Florida, Orlando, FL 32816, USA; carmen.giurgescu@ucf.edu

**Keywords:** preterm birth, vitamin D, pregnancy, adverse outcomes

## Abstract

Vitamin D deficiency (25 (OH)D < 20 ng/mL) is a modifiable risk factor that has been associated with an increased risk of preterm birth (PTB) (<37 weeks gestation). Black women are at a high risk for vitamin D deficiency due to higher melanin levels. Vitamin D sufficiency may be protective against PTB risk in Black women. Black participants between 8 and 25 weeks of gestation were included in this nested case–control study. The sample consisted of women who had either PTBs (n = 57) or term births, were selected based on maternal age compared to those who had PTBs (n = 118), and had blood samples available between 8 and 25 weeks of gestation. The women completed questionnaires about depressive symptoms and smoking behavior and had blood collected to determine their vitamin D levels. Gestational age at birth, hypertensive disorders, and body mass index (BMI) were collected from the medical records. The odds of PTB were increased by 3.34 times for participants with vitamin D deficiency after adjusting for hypertensive disorders of pregnancy and depressive symptoms. Vitamin D assessment and supplementation may be an important intervention for preventing PTB in pregnant Black women.

## 1. Introduction

In 2021, the disparity of preterm birth (PTB) among non-Hispanic Black women was doubled (14.8%) when compared to non-Hispanic White women (9.5%), a persistent increase in risk over decades [1,2]. Higher socioeconomic status, maternal education, and insurance status have not been shown to improve the risk of PTB for Black women in large epidemiological studies [3,4]. No effective interventions have been identified to mitigate the risk of PTB for Black women. Vitamin D deficiency (25 (OH)D < 20 ng/mL) is a modifiable risk factor that has been associated, albeit inconsistently, with an increased risk of PTB [5,6,7]. Black women are at high risk for vitamin D deficiency [8] due to increased melanin which protects against harmful ultraviolet rays but inhibits vitamin D absorption and metabolism [9,10]. According to the National Health and Nutrition Examination Survey (NHANES), 72% of the non-Hispanic Black population were deficient in vitamin D [8]. Due to increased maternal and fetal use of vitamin D, the Endocrine Society considers pregnancy itself a risk factor for vitamin D deficiency [11] and encourages higher supplementation (1500–2000 IUs daily) than the Institute of Medicine (IOM) guidelines, which only recommend the standard 400 IUs daily [12]. In addition, the Endocrine Society defines vitamin D sufficiency as 25(OH)D > 30 ng/mL, whereas the Institute of Medicine defines vitamin D sufficiency as 25(OH)D ≥ 20 ng/mL [11].

Vitamin D deficiency has been related to depressive symptoms and an increased risk of PTB [13,14,15,16]. Pregnant women who are deficient in vitamin D are at a higher risk for depressive symptoms both during pregnancy and the postpartum period [13,17,18,19,20]. Prior work also showed that pregnant Black women who have more depressive symptoms are at a higher risk of PTB than Black women with no depressive symptoms [21,22,23]. The conversion of 25(OH)D into 1, 25dihydroxyvitaminD (1,25(OH)_2_D) facilitates immune function through a vitamin D receptor (VDR)-mediated transcription of a diverse array of cells at the fetal and maternal interface, with anti-inflammatory and tolerogenic principal effects [24,25,26]. The causes of PTB are multifactorial, but they are categorized as either medically indicated or spontaneous [27]. One of the most common reasons for medically indicated PTB is preeclampsia [27]. The spontaneous causes of PTB are due to premature labor and/or maternal infection and inflammation at the chorion–placental interface [28]. Both medically indicated and spontaneous PTBs can potentially be mitigated via vitamin D due to the immunomodulatory impact vitamin D has on infection and inflammation during pregnancy. Other factors that also contribute to the risk of PTB include obesity [29,30], prenatal smoking [31,32], and any history of hypertension, either pre-pregnancy or gestational [33,34]. Thus, the purpose of this study was to explore whether vitamin D deficiency in early pregnancy increased the risk of PTB in later pregnancy in a cohort of Black women while adjusting for other confounders.

## 2. Materials and Methods

### 2.1. Study Design and Participants

The data for this nested case–control study were obtained from a subset of the Biosocial Impact on Black Births (BIBB) study, which has been published [35,36]. The BIBB study was an NIH-funded, prospective mixed-methods study that recruited pregnant Black women from two different geographic sites in the United States located in the Midwest region. The data included in these analyses were collected from women recruited between December 2017 and March 2020, when the study was placed on hold due to the COVID-19 pandemic.

Women were included in the BIBB study if they self-described as being African American or Black, were 18–45 years of age, had a singleton pregnancy, were enrolled between 8 and 29 weeks of gestation, and could read and write English. Eligible women were approached by the research staff about the study and invited to participate. Women who agreed to participate completed an informed consent process. The participants completed questionnaires on an electronic device, and blood samples were collected from them via venipuncture two or three times during their pregnancy. The participants were compensated with store gift cards for each questionnaire and biological sample collected up to a maximum of USD 175. The research staff collected birth (e.g., PTB) and medical history data (e.g., hypertensive disorders of pregnancy) from maternal medical records.

Women with PTB were included in the analysis if they completed the questionnaires and had blood samples collected between 8 and 25 weeks of gestation. The gestational age range of 8–25 weeks was selected to include as many women with PTB as possible. A subsample of 57 women with PTB had both questionnaire responses and blood samples collected between 8 and 25 weeks of gestation. The maternal age for women with PTB ranged from 18 to 41 years of age. Women who had a term birth (defined as ≥37 weeks of gestation) were included in the analysis based on the availability of data from the questionnaires and the blood samples collected between 8 and 25 weeks of gestation, and they were selected using the same quartiles of maternal age as the women with PTB. A subsample of 264 women with term birth had questionnaire data and blood available at 8–25 weeks of gestation, and they had similar age ranges as the women with PTB. We randomly selected 118 women from that subsample. We selected 118 women with term birth, not a matched set of controls, to maintain an approximate 2:1 ratio of term birth to PTB and to maximize power within the resources available for the laboratory assays. Since this study was a nested control–cohort study, we did not match any demographic data other than maternal age, but statistical comparisons are provided in Table 1 to ensure the PTB and term groups were not significantly different with respect to demographic variables.

### 2.2. Variables and Instruments

Maternal characteristics. Demographic data were collected via self-reporting and included maternal age, level of education, marital status, employment, and annual household income. Maternal smoking status was determined based on self-reports of the frequency of smoking cigarettes and e-cigarettes. Any smoking during pregnancy was coded as “yes”, and not smoking was coded as “no”.

Maternal Body Mass Index (BMI). Maternal weight and height were measured by prenatal clinic staff at the first prenatal visit if weight was available prior to 14 weeks of gestation and retrieved from the electronic medical records. The prenatal body mass index (BMI, weight(kg)/height(m^2^)) was calculated using IOM guidelines, and obesity was defined as a BMI of ≥ 30 m^2^ and analyzed as a categorical variable (obese = BMI of ≥ 30 m^2^; non-obese = BMI < 30 m^2^) [37].

Depressive symptoms. The Center for Epidemiologic Studies Depression Scale (CES-D) was used for measuring symptoms of depression. The CES-D is a 20-item instrument on a 4-point scale (ranging from 0 = rarely to 3 = most of the time) with a seven-day recall period and a total score ranging from 0 to 60 [38]. A higher score represents more symptoms of depression, with a cut-off point of ≥23 being highly correlated with a clinical diagnosis of depression in pregnancy [39].

Hypertensive disorders of pregnancy (HDP). Retrospective medical record abstraction was conducted to identify participants with any diagnosis of hypertensive disorder in pregnancy (HDP), including preeclampsia, eclampsia, gestational hypertension, pre-pregnancy hypertension, and superimposed preeclampsia (pre-pregnancy hypertension with the development of preeclampsia). The variable was binary, with 0 representing no HDP diagnosis and 1 representing any HDP diagnosis.

Plasma total 25(OH)D. Blood was collected via venipuncture in 6 mL EDTA tubes between 8 weeks and 25 weeks of gestation. The blood samples were kept on ice or in a refrigerator until processing and were centrifuged (1600× *g* 15 min at 4° Celsius) within 2 h of collection to separate the plasma from red blood cells. A total of 1 mL of plasma was pipetted and aliquoted into 1.5 mL microcentrifuge tubes and frozen at −80 °C in freezers at two different data collection sites. The plasma was analyzed at Heartland Assays using liquid chromatography–mass spectrometry (LC/MS). Intra- and inter-assays of the Coefficient of Variance (CV) were <5%. Vitamin D deficiency was defined as 25(OH)D < 20 ng/mL based on the IOM guidelines [40].

### 2.3. Statistical Analysis

Data were analyzed using IBM SPSS Statistics 28. Descriptive statistics for the variables in the analysis included frequencies(n) and percentages for categorical variables and means and standard deviations for continuous variables. Independent samples *t*-tests and χ^2^ tests were used to determine whether there were statistically significant differences in maternal characteristics between women with PTB and women with term birth. Bivariate analyses were conducted with each potential predictor variable (vitamin D, depressive symptoms, HDP, BMI, and prenatal smoking) and PTB unadjusted to screen for potential issues with multicollinearity. Binary logistic regressions were then performed to predict the odds of PTB depending on vitamin D status, controlling for depressive symptoms, HDP, BMI, and prenatal maternal smoking. Logistic regression was selected for the analysis because the gestational age in weeks was non-normally distributed and logistic regression provides interpretable estimates of the probability of having a PTB or term birth. Due to slight differences in the average gestational age at blood draw and to increase confidence in the logistic regression estimates, propensity scores were calculated based on the gestational age at the time of blood draw and maternal age. As plasma 25(OH)D changes throughout gestation [41], propensity weights were then applied to the final logistic regression model to account for the potential confounding effects of maternal age and gestational age at blood draw on the relationship between vitamin D status and PTB.

## 3. Results

### 3.1. Descriptive Characteristics of the Sample

Characteristics of the sample by term birth and vitamin D status are shown in Table 1. The independent samples *t*-tests showed no significant differences in maternal age or gestational age at blood draw between (1) women with PTB and women with term birth and (2) women with vitamin D deficiency and women with vitamin D sufficiency. Categorical variables were assessed using bivariate tables and χ^2^ tests. The results of the χ^2^ analysis also showed no significant differences between the groups.

### 3.2. Binary Logistic Regressions

Binary logistic regressions were performed to predict the odds of having PTB depending on vitamin D sufficiency while controlling for depressive symptoms and HDP as the only remaining confounders, using the epidemiologic rule that if the relative change in the odds ratio (OR) after an adjustment for a potential confounder is ~10% or greater, it should be kept in the adjusted model [38]. Obesity did not correlate with PTB as an outcome. Prenatal smoking did significantly predict PTB, but when added to the binary logistic regression, the odds ratio changed by only 0.65%. Therefore, depressive symptoms, as measured via the CES-D and HDP, remained in the adjusted model. All models were statistically significant based on each model’s χ^2^ output.

**Figure 1:**
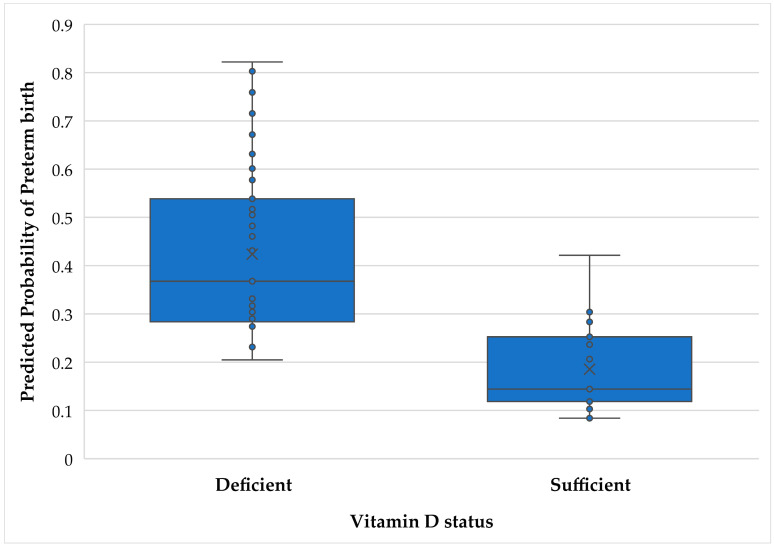
Predicted probability of PTB by Vitamin D sufficiency, adjusted for other predictors.

Table 2 includes the results of the binary logistic regression analyses predicting PTB. The unadjusted model included only vitamin D as a predictor of PTB. The results showed that women with vitamin D deficiency were 2.80 times as likely to have a PTB than women with vitamin D sufficiency (OR = 2.80, 95% CI: 1.40, 5.59, *p* ≤ 0.01). The unweighted adjusted model included vitamin D along with CES-D scores ≥ 23 and any HDP diagnosis. Figure 1 illustrates the adjusted probability of PTB compared with term birth by vitamin D status. Vitamin D remained a significant predictor of PTB in the adjusted model, with a slightly stronger odds ratio (OR = 3.00, 95% CI: 1.47, 6.15, *p* ≤ 0.01). Vitamin D showed a slightly weaker effect (OR = 2.74, 95% CI: 1.35–5.54) after propensity score weights were applied to account for maternal age, gestational age at blood draw, and confounders (depressive symptoms and HDP). However, the overall results are similar among all three logistic regression models.

## 4. Discussion

Plasma vitamin D deficiency during pregnancy was a significant predictor of PTB among a cohort of Black women in our study after controlling for depressive symptoms and HDP. Although we did not limit PTBs to spontaneous PTBs, vitamin D deficiency was more strongly associated with PTB after controlling for HDP, the leading cause of non-spontaneous (medically indicated) PTB. These findings support the need to develop approaches for the supplementation of vitamin D for women who have vitamin D deficiency. Research into how supplementation can be utilized more effectively and efficiently is needed, especially among Black women who are both the most vulnerable to vitamin D deficiency and have the highest rates of PTB [2,42,43].

The results of our study are consistent with other studies that included a significant percentage of Black women [5,6,43]. Several meta-analyses and systematic reviews that were published in the last 5 years have shown a significant association between vitamin D deficiency and PTB [5,44,45]. However, there are still inconsistencies and heterogeneities of the studies included in the review as the studies used different cut-offs for measuring vitamin D deficiency, with some studies using the Institute of Medicine’s guidelines of <20 ng/mL and some using the Endocrine Society’s cutoff value of <30 ng/mL for vitamin D insufficiency [11,12]. Further, many of the studies included in those meta-analyses and systematic reviews did not have a large population of Black women, which is a unique contribution of our study. In a multiethnic cohort of 3453 pregnant women, 28.2% of the women who had a PTB (n = 1127) were non-Hispanic Black; however, non-Hispanic Black women comprised only 12.8% of the sample [43]. Women who had serum 25(OH)D < 20 ng/mL were 2.1 times more likely to have a PTB at <34 weeks after adjusting for maternal race, pre-pregnancy BMI, and other significant covariates (OR 2.1; 95% CI 1.3, 3.6) [43]. In another multi-ethnic cohort (n = 2629) with 46% of the women identifying as non-Hispanic Black, 66% of the spontaneous PTBs occurred among non-Hispanic Black women [46]. There was no association of serum 25(OH)D with PTB among White women, but a 30% increased risk of spontaneous PTB < 35 weeks was found in non-White pregnant women with vitamin D deficiency (RR 0.73; 95% CI 0.59–0.89). Bodnar et al. (2014) defined spontaneous PTB as <35 weeks of gestation compared with our study, which used a definition of PTB < 37 weeks of gestation. [46] which is the clinical standard for defining PTB [28]. They also limited their definition of PTB to those who delivered spontaneously due to preterm labor with intact membranes or the premature rupture of membranes between 26 and <35 weeks of gestation [46].

Our study was unique in that vitamin D deficiency remained predictive of PTB and improved the odds ratio after HDP and depressive symptoms were adjusted for. There is also evidence that vitamin D deficiency increases the risk of preeclampsia as in one randomized control trial, early and late pregnancy vitamin D levels of >30 ng/mL upon entry to a clinical trial reduced the risk of preeclampsia (adjusted OR = 0.28; 95% CI, 0.10–0.96) [47]. Vitamin D may play an important role in inflammation, which could explain its relationship with increased risks of PTB and preeclampsia in women who have vitamin D deficiency [48,49].

One result from our study that conflicts with other studies which included large numbers of pregnant Black women [13,15,17] is that pregnant Black women with vitamin D deficiency defined as <20 ng/mL did not have more significant symptoms of depression; however, when included in the adjusted logistic regression model with HDP, it did increase the OR for PTB. In contrast, Accortt et al. (2018) collected serum in early and late pregnancy as well as during the postpartum period from a cohort of multi-ethnic pregnant women (18% non-Hispanic Black) and found an association between women with vitamin D deficiency and higher levels of depressive symptoms across pregnancy [17]. Women who had vitamin D deficiency also had a higher relative risk for adverse pregnancy outcomes which included preeclampsia, PTB, and low birth weight [17]. This is significant since increased depressive symptoms and vitamin D deficiency have both been associated with an increased risk of PTB [50,51].

The insidious and multi-factorial nature of the causes of PTB makes developing treatment for or potentially preventing PTB challenging. However, our findings support vitamin D supplementation as a possible intervention that could potentially reduce the risk of PTB. However, observational studies of risk factors for PTB are not the gold standard for establishing whether an intervention utilizing vitamin D supplementation will be effective. Multiple randomized controlled trials (RCTs) have been conducted to assess whether vitamin D supplementation can improve the risk of PTB or pre-eclampsia; however, the results have been conflicting [5,44,52,53]. Heterogeneous doses of vitamin D supplementation usage and flawed study designs have plagued the supplementation trials thus far. For example, some RCTs do not exclude pregnant women with vitamin D sufficiency, which makes supplementation unlikely to have an impact on PTB or preeclampsia because the inclusion of women with vitamin D sufficiency inflates type I error. RCTs have also rarely used a sufficient treatment/supplementation dose (i.e., 400 IU) [7,41,54]. The timing of the vitamin D supplementation and a focus on populations that have severe vitamin D deficiency as defined by the Endocrine Society as 25(OH)D < 12.5 ng/mL may also be critical factors in decreasing the risk of PTB. There is some evidence that vitamin D sufficiency in the mid-second trimester and early third trimester may be beneficial in decreasing the risk of PTB [6,55]. Interestingly, other populations that have vitamin D deficiency, such as persons from India [56] and the Middle East [57], demonstrated a decreased risk of PTB with vitamin D supplementation of 4000 IUs or greater. Therefore, ensuring that women achieve adequate levels of vitamin D during pregnancy is an important determinant of decreasing the risk of PTB [10,48,58].

This study highlights the importance of reaching sufficient levels of vitamin D during pregnancy, specifically among Black women. In addition, there is sufficient evidence that there are vitamin-D-binding protein single-nucleotide polymorphisms (SNPs) and other vitamin-D-related gene variants that can significantly impact vitamin D metabolism and may be associated with PTB [59,60,61]. People of African ancestry more commonly have the homozygous 1F allelic variant of the VDBP gene and, based on vitamin D supplementation studies, people with this variant need almost double the recommended supplementation to achieve a normal vitamin D level [59,62]. This is clinically significant and can explain why there have been conflicting results from vitamin D supplementation trials. Past research has not incorporated vitamin-D-related gene variants and how they might help achieve sufficient vitamin D levels and consequently impact outcomes such as PTB. We have typically used a one-size-fits-all approach to nutrient supplementation, but perhaps we need to incorporate a precision medicine approach and individualized supplementation based on diet and genotypes to achieve improved outcomes. The results from this study show that vitamin D status in Black women could be an important biomarker for further study.

Strengths and Limitations. One of the main strengths of this study is that the sample comprised Black women only. Black women are at the greatest risk for both vitamin D deficiency [8] and PTB [2]. More needs to be learned about the unique pathways to PTB that impact Black women and contribute to the continuing health disparity of the risk of PTB experienced by Black women. Many other studies that examined the association of vitamin D deficiency with the risk of PTB did not include a predominantly Black population and could not determine the effects in this at-risk population [7,42].

The limitations of this study include its relatively small sample size and the lack of dietary data for the participating women. This was a secondary analysis of a prenatal cohort of Black women in which the focus was not on nutrition; thus, dietary data were not collected. The most common sources of dietary vitamin D and calcium are fatty fish and foods fortified with vitamins such as cow’s milk, soy milk, orange juice, and egg yolks [40,41]. Dietary intake of vitamin D and calcium is fairly minimal generally but may be especially more so in Black populations given the high prevalence of lactose intolerance [63]. Therefore, supplementation is likely to be required if vitamin D deficiency is to be addressed for pregnant Black women.

Another limitation of the study was that there was such a large range of gestational age at blood draw in the sample of participants (8–25 weeks gestation) which is significant since there is evidence to show that 25(OH)D levels can change depending on the trimester of pregnancy [48]. We attempted to adjust for that by creating the propensity score weights to help control for maternal age and gestational age at blood draw. As noted in Table 1, there was no statistical difference in gestational age at blood draw between the term and PTB group and was close to being statistically different in the vitamin D sufficient and deficient group.

## 5. Conclusions

We found that plasma vitamin D deficiency during pregnancy was a significant predictor of PTB among a cohort of Black women. Vitamin D supplementation during pregnancy may be an important determinant in decreasing the risk of PTB among Black women. RCTs are needed to examine whether vitamin D supplementation decreases the risk of PTB among these women and which dose of supplementation is more likely to provide the greatest improvements in pregnancy outcomes, especially PTB. Future research must focus on understanding methods for maximizing the benefits of improving vitamin D status in pregnancy and whether this indicates a higher supplementation dose or initiating supplementation prior to pregnancy to achieve healthy pregnancy outcomes. More research must also be conducted to understand how the intersection between genetics and environment in relation to vitamin D increases the risk of PTB. It will be important to evaluate the molecular mechanisms that sufficient vitamin D status can make on pregnancy outcomes such as PTB. Lastly, more studies need to be conducted to elucidate the molecular pathway through which calcitriol exerts its maximal effect on the immune system and how it can prevent poor outcomes such as PTB.

## Figures and Tables

**Table 1:** Characteristics of the sample (n = 175).

Characteristics	Preterm Birth (n = 57)	Term Birth (n = 118)	*p* Value	Vitamin D Deficiency (n = 74)	Vitamin D Sufficiency (n = 101)	*p* Value
	Mean ± SD					
Maternal Age (years)	27.77 ± 5.91	27.14 ± 5.83	0.501	27.66 ± 5.96	26.91 ± 5.70	0.398
Gestational Age at Blood Draw (weeks)	14.21 ± 4.88	13.93 ± 4.07	0.692	13.48 ± 4.07	14.77 ± 4.61	0.051
	Frequency (%)					
Annual Household Income			0.230			0.879
≤US 10,000	27 (47.4%)	49 (41.5%)		46 (45.5%)	30 (40.5%)	
USD 10,000–19,999	10 (17.5%)	17 (14.4%)		14 (13.9%)	13 (17.6%)	
USD 20,000–29,999	14 (24.6%)	24 (20.3%)		22 (21.8%)	16 (21.6%)	
≥USD 30,000	6 (10.5%)	28(23.7%)		19 (18.8%)	15 (20.3%)	
Level of Education			0.294			0.685
Less than high school	7 (12.3%)	15 (12.7%)		12 (11.9%)	10 (13.5%)	
High school or GED	27 (47.4%)	53 (44.9%)		49 (48.5%)	31 (41.9%)	
>High school education	23 (40.3%)	50 (42.4%)		40 (39.6%)	33 (44.6%)	
Marital status			0.615			0.712
Married/live with partner	24 (42.1%)	45 (38.1%)		41 (40.6%)	28 (37.9%)	
Work Status			0.355			0.355
Currently working	31 (54.4%)	55 (46.6%)		48 (47.5%)	38 (51.4%)	
Insurance			0.676			0.474
Medicaid	38 (66.7%)	71 (60.2%)		63 (62.4%)	46 (62.2%)	
Medicare	6 (10.5%)	11 (9.3%)		12 (11.9%)	5 (6.8%)	
Medicaid + Medicare	4 (7.0%)	14 (11.9%)		8 (7.9%)	10 (13.5%)	
Private or other	9 (15.8%)	22 (18.6%)		18 (17.8%)	13 (17.6%)	
CES-D scores ≥ 23	13 (22.8%)	31 (26.3%)	0.621	28 (27.7%)	16 (21.6%)	0.358
HDP diagnosis	28 (49.1%)	32 (27.1%)	0.004 *	35 (34.7%)	25 (33.8%)	0.905
Obese (BMI ≥ 30 kg/m^2^)	30 (52.6%)	60 (50.8%)	0.825	57 (56.4%)	33 (44.6%)	0.122

Continuous variables are presented as means ± standard deviations. Values for categorical variables are presented in frequency (%). CESD, Center for Epidemiological Studies Depression Scale; HDP, hypertensive disorders of pregnancy. Obesity is based on the body mass index (kg/m^2^) at the first prenatal visit. * *p* < 0.01

**Table 2:** Binary logistic regression predicting the odds of preterm birth (n = 175).

	Preterm Birth					
Variables	Vitamin D Unadjusted Odds Ratio		Unweighted Adjusted Odds Ratio ^1^		Weighted Adjusted Odds Ratio ^1^	
	OR	95% CI	OR	95% CI	OR	95% CI
Vitamin D deficiency (25(OH)D ≤ 20 ng/mL)	2.80 **	1.40–5.59	3.00 **	1.47–6.15	2.74 **	1.35–5.54
Model χ^2^	7.83 *		9.09 *		7.83 *	
−2 log-likelihood	211.75		202.66		237.00	

* *p* ≤ 0.05, ** *p* ≤ 0.01, ^1^ Adjusted for the CES-D (Center for Epidemiological Studies Depression Scale) and HDP (hypertensive disorders of pregnancy).

## Data Availability

Not applicable.
